# Educational needs and career development of young epileptologists in Italy

**DOI:** 10.1002/epi4.12888

**Published:** 2024-02-22

**Authors:** Carlotta Spagnoli, Maddalena Duca, Veronica Pelliccia, Jacopo Lanzone, Silvia Masnada, Daniela Chiarello, Tommaso Lo Barco, Fedele Dono, Bruna Nucera

**Affiliations:** ^1^ Child Neuropsychiatry Unit, Pediatric Department Santa Maria Nuova Hospital, Azienda USL‐IRCCS di Reggio Emilia Reggio Emilia Italy; ^2^ Child Neuropsychiatry Unit Ospedale Civile di Macerata Macerata Italy; ^3^ "Claudio Munari" Epilepsy Surgery Centre Azienda Socio‐Sanitaria Territoriale Grande Ospedale Metropolitano Niguarda Milan Italy; ^4^ Neurorehabilitation Department of the Milano Institute Istituti Clinici Scientifici Maugeri IRCCS Milan Italy; ^5^ Child Neurology Unit Buzzi Children's Hospital Milan Italy; ^6^ Child Neuropsychiatry Department, Epilepsy Center C. Poma Hospital Mantova Italy; ^7^ Department of Neuroscience, Imaging and Clinical Science "G. D'Annunzio" University of Chieti‐Pescara Chieti Italy; ^8^ Department of Neurology Hospital of Merano (SABES‐ASDAA) Merano Italy; ^9^ Paracelsus Medical University Salzburg Austria

**Keywords:** career development, educational needs, ILAE, LICE, survey, Young Epilepsy Section

## Abstract

**Objective:**

The Education and Career Task Force of the Young Epilepsy Section‐Italy focuses on educational and career development needs of young Italian epileptologists. Two surveys were developed (pre‐ and post COVID‐19 pandemic) in order to identify the needs of members of the Lega Italiana Contro l'Epilessia under 40 years of age.

**Methods:**

The first was distributed during the 42nd National Congress (Rome, June 5–7, 2019); the second during the 45th National Congress (Padova, June 8–10, 2022) and subsequently by e‐mail until July 9, 2022. Data from the 2019 survey were analyzed descriptively. Data from the 2022 survey were further analyzed with Pearson's chi‐square test to establish if gender, field of clinical practice, and professional role were associated with different needs.

**Results:**

Sixty surveys were completed in 2019 and 69 in 2022. Attendance to courses and congresses as the preferred way to keep medical knowledge updated reduced between 2019 and 2022. The reason was different between trainees (mostly elevated costs) and early‐career consultants (mostly organizational issues) (*p* = 0.005). The main needs for improvement also diverged: trainees indicated differential diagnosis and diagnostic approach to the first seizure while consultants indicated diagnostic approach to genetic epilepsies (*p* = 0.004); in the genetic field, priority needs were selection of genetic investigations for trainees versus genotype–phenotype correlations for consultants (*p* = 0.022). The field of practice (pediatric vs. adult) also impacted on the main needs for improvement that is, acquisition of expertise in neuroradiology and drug therapy for pediatric versus genetics for adult neurology trainees or consultants (*p* = 0.018); in the clinical area, differential diagnosis and approach to the first seizure versus status epilepticus (*p* = 0.027); in the genetic field, precision medicine versus genotype–phenotype correlations (*p* = 0.034). No differences were found based on gender.

**Significance:**

The surveys identified different needs based on professional role and discipline.

**Plain Language Summary:**

The Education and Career Task Force of the Young Epilepsy Section‐Italy (YES‐I) launched two surveys among young Italian epileptologists. Our research shows that the educational and professional needs of young Italian epileptologists vary based on their job role and field of practice, but not on gender. Their preference for on‐site congresses and courses reduced after the pandemic, and the main reason is linked to financial constraints for trainees and to organizational issues for consultants. The main expectation toward YES‐I is to receive support for education and career development. Thus, we collected useful suggestions on how to organize our future YES‐I activities.

Key Points

- The Education and Career Task Force of the Young Epilepsy Section‐Italy (YES‐I) launched two surveys among young Italian epileptologists.
- Attendance to courses and congresses as the preferred way to keep medical knowledge updated reduced after COVID‐19 pandemic.
- The main needs for improvement differ between consultants and trainees, and according to field of practice (adult vs. pediatric).
- The most valued YES‐I mission and activities also differ according to job position and field of practice.
- Gender of responders did not influence educational and career development needs.

## 1 INTRODUCTION

The first meeting of the Young Epilepsy Section (YES) of the International League Against Epilepsy (ILAE) took place at the YES kick‐off workshop on May 12–13, 2018, in London, UK. YES is a worldwide organization of young people in the early stages of their career focused on epilepsy care and/or research. Its main objectives are to improve professional developments of young people working on epilepsy and expand their involvement across ILAE globally, in order to shape the new generation of epileptologists (https://www.ilae.org/about‐ilae/topical‐commissions/yes/young‐epilepsy‐section‐yes/about‐us, last visited on April 27, 2023).

On January 26, 2019, the first meeting of the YES Italian branch (YES‐I) took place in Rome, Italy. YES‐I include young (i.e., under 40 years) members of the “Lega Italiana Contro l'Epilessia (LICE),” Italian chapter of the ILAE. Within YES‐I, the Education and Career Development Task Force was created with the aim to disseminate courses and congresses, promote educational and training experiences for students, trainees, and young consultants, advertise job opportunities or doctoral bursaries, promote mentoring activities, and organize educational meetings (https://www.lice.it/LICE_ita/YES‐I/YES‐I_presentazione.php, last visited on April 27, 2023).

In order to identify the educational needs and the expectations on career development opportunities to be delivered by the YES‐I Education and Career Development Task Force, a first survey was distributed in 2019 (“pre‐pandemic”) and a second one in 2022 (“post‐pandemic”).

## 2 METHODS

### 2.1 2019 survey

The first survey was distributed on paper to LICE members up to 40 years of age during the 42nd National Congress of LICE (Rome, June 5–7, 2019). Its main objective was to select topics to organize a dedicated session during the following LICE Congress to be held in 2020, then held virtually due to the COVID‐19 pandemic.

The survey was composed of two sections: the first one focusing on demographic data, while the second one concerned the educational needs with nine multiple choice questions and one open question. In the second section, more than one answer was allowed for each question.

### 2.2 2022 survey

The second survey was launched by e‐mail until 9/7/2022 to all YES‐I members and proposed via QR code during the 45th National LICE Congress (Padua, June 8–10, 2022). It was composed of a first part reproducing the 2019 questionnaire, and a second part adding some questions to explore the preferred topics for a congress dedicated to YES‐I members, and to understand the impact of the COVID‐19 pandemic in training, education, and career development.

### 2.3 Statistical analysis

Surveys were filled‐in freely and anonymously. Data from the 2019 survey were processed using Microsoft Excel. We performed a descriptive analysis, by calculating absolute numbers and percentages for each reply. Data from the 2022 survey were further analyzed with Pearson's chi‐square test in order to establish if gender, field of clinical practice (child neuropsychiatry, adult neurology, or both), and professional role (consultant, trainee, PhD student/research fellow, or other) were associated with different educational or career development needs.

We extrapolated the pseudonymized list of YES members for years 2019 and 2022 and compared demographic data with those of the surveys, by performing Pearson's chi‐square test.

In both cases, a *p* ≤ 0.05 was considered as statistically significant.

## 3 RESULTS

### 3.1 General survey results

**FIGURE 1:**
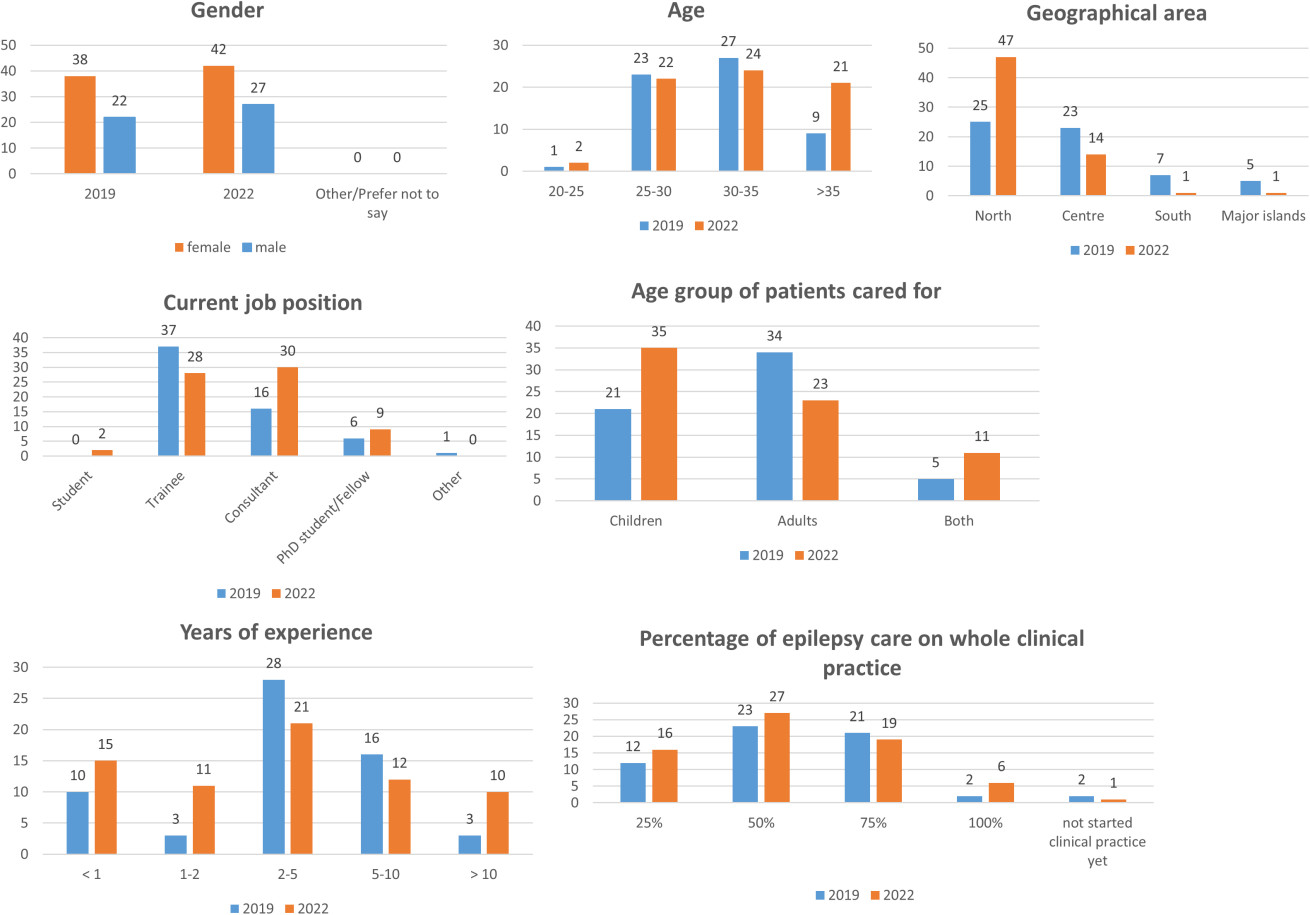
Demographic data of responders, compared data (2019 vs. 2022).

**FIGURE 2:**
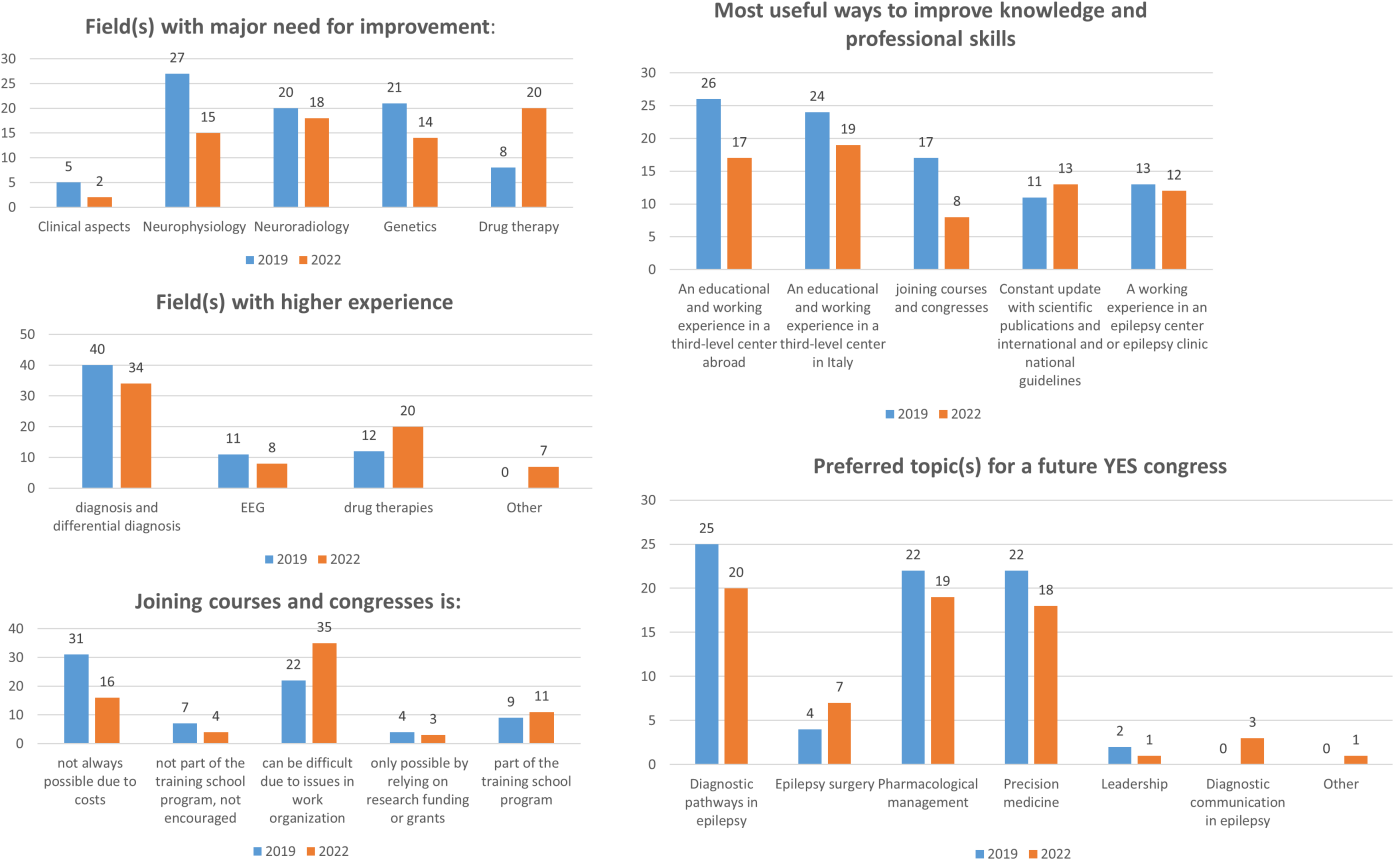
Educational and career development needs, compared data (2019 vs. 2022).

**FIGURE 3:**
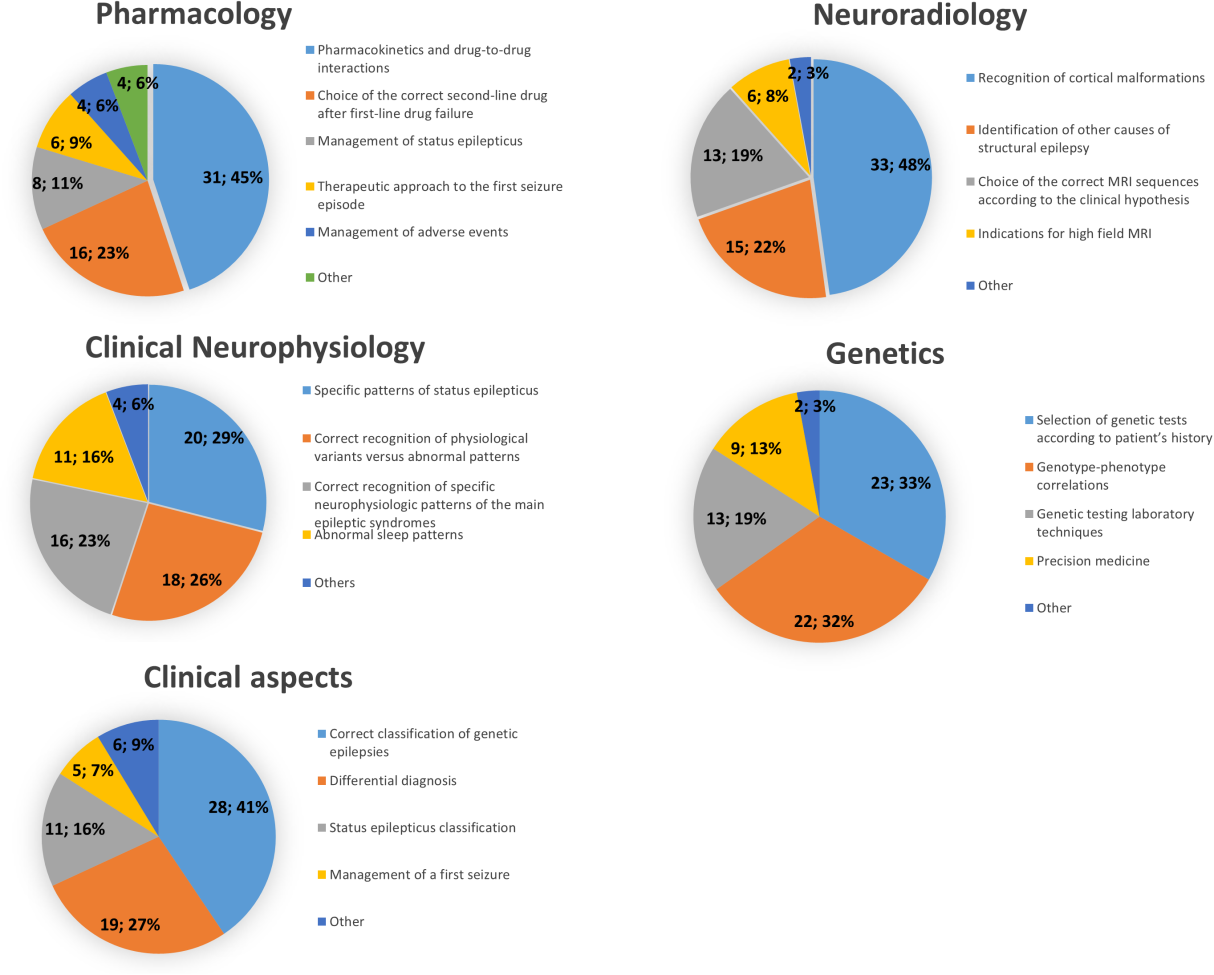
Major needs of improvements within each proposed field of epileptology, compared data (2019 vs. 2022).

**FIGURE 4:**
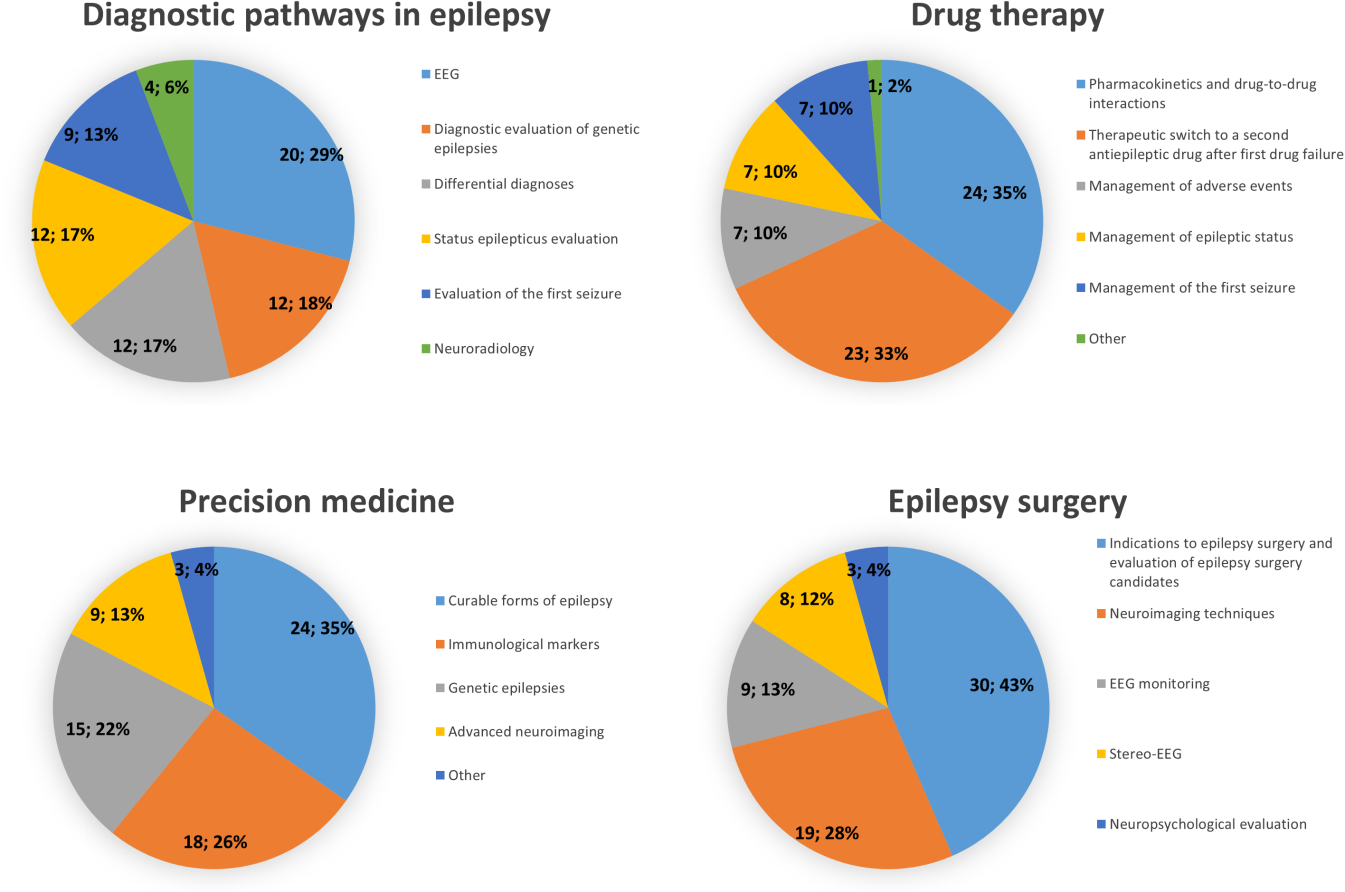
Preferred topics for a future YES‐I congress within each of the proposed fields of epileptology (2022 survey).

**FIGURE 5:**
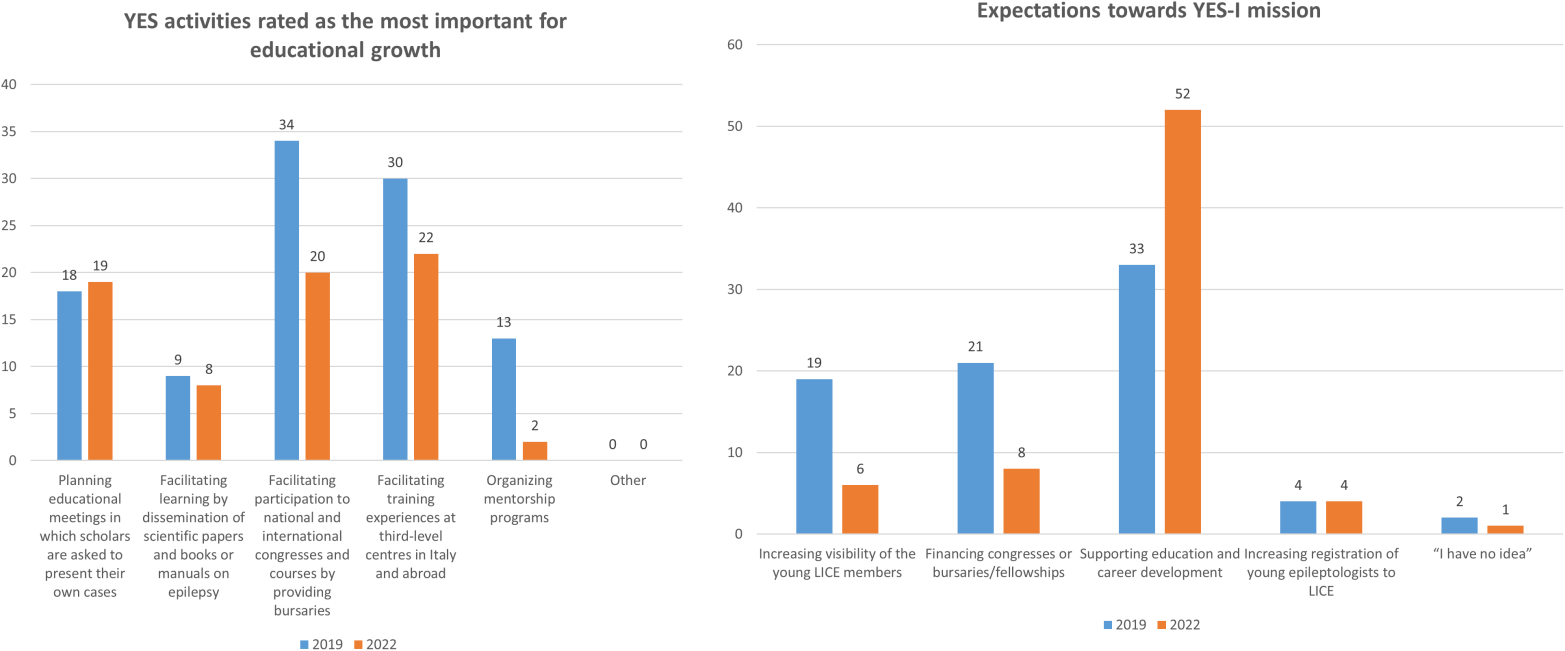
Expectations toward YES‐I role and activities, compared data (2019 vs. 2022).

Sixty YES‐I members participated to the survey in 2019. This corresponds to a response rate of 15% among LICE members up to 40 years of age (*n* = 398). In 2022, we collected 69 replies, corresponding to a response rate of 13.4% (69/512). All participants replied to 100% of the questions. A summary of the main results will be reported in the following. The complete surveys results can be found in Table 1 and in Figures 1, 2, 3, 4, 5.

**TABLE 1:** Survey results (demographic data, educational, and career development needs), compared data (2019 vs. 2022).

	2019 *n* (%)	2022 *n* (%)
Gender		
Female	38/60 (63.3%)	42/69 (60.9%)
Male	22/60 (36.7%)	27/69 (39.1%)
Other/prefer not to say	0	0
Age		
20–25	1/60 (1.7%)	2/69 (2.9%)
25–30	23/60 (38.3%)	22/69 (31.9%)
30–35	27/60 (45.0%)	24/69 (34.8%)
>35	9/60 (15%)	21/69 (30.4%)
Geographical area		
North	25/60 (41.7%)	47/69 (68.1%)
Central	23/60 (38.3%)	14/69 (20.3%)
South	7/60 (11.7%)	7/69 (10.1%)
Major islands	5/60 (8.3%)	1/69 (1.5%)
Current professional position		
Student	0	2/69 (2.9%)
Trainee	37/60 (61.6%)	28/69 (40.6%)
Consultant	16/60 (26.7%)	30/69 (43.5%)
PhD student/Fellow	6/60 (10.0%)	9/69 (13%)
Other	1/60 (1.7%): biologist	0
Age group of patients cared for		
Children	21/60 (35.0%)	35/69 (50.7%)
Adults	34/60 (56.7%)	23/69 (33.3%)
Both	5/60 (8.3%)	11/69 (16.0%)
Years of clinical experience in managing epilepsy		
<1	10/60 (17.0%)	15/69 (21.7%)
1–2	3/60 (5.0%)	11/69 (15.9%)
2–5	28/60 (46.6%)	21/69 (30.4%)
5–10	16/60 (26.6%)	12/69 (17.4%)
>10	3/60 (5.0%)	10/69 (14.6%)
Percentage of epilepsy management on the whole clinical practice		
25%	12/60 (20.0%)	16/69 (23.2%)
50%	23/60 (38.3%)	27/69 (39.1%)
75%	21/60 (35%)	19/69 (27.5%)
100%	2/60 (3.3%)	6/69 (8.7%)
I haven't worked clinically on epilepsy yet	2/60 (3.3%)	1/69 (1.5%)
Field(s) with major need for improvement^a^		
Clinical neurophysiology	27/81 (33.3%)	15/69 (21.7%)
Genetics	21/81 (25.9%)	14/69 (20.3%)
Neuroradiology	20/81 (24.7%)	18/69 (26.1%)
Drug therapy	8/81 (9.9%)	20/69 (29.0%)
Clinical aspects	5/81 (6.2%)	2/69 (2.9%)
Field(s) with higher experience^a^		
Diagnosis and differential diagnosis	40/63 (63.5%)	34/69 (49.3%)
EEG	12/63 (19.0%)	20/69 (29.0%)
Pharmacological therapies	11/63 (17.5%)	8/69 (11.6%)
Other	NA	7/69 (10.1%)
Participation to courses and congresses^a^		
Is not always possible due to their costs can be difficult to attend due to work organization restraints	31/73 (42.5%) 22/73 (30.1%)	16/69 (23.2%) 35/69 (50.7%)
Is part of their scheduled educational program	9/73 (12.3%)	11/69 (15.9%)
Is not considered as part of their training school educational program and thus is not encouraged	7/73 (9.6%)	4/69 (5.8%)
It is possible by relying on research funding or grants	4/73 (5.5%)	3/69 (4.3%)
Preferred ways to improve knowledge and professional skills^a^		
An educational and working experience in a third‐level center abroad	26/91 (28.6%)	17/69 (24.6%)
An educational and working experience in a third‐level center in Italy	24/91 (26.4%)	19/69 (27.5%)
Ongoing education by attending courses and congresses	17/91 (19.0%)	8/69 (11.6%)
A working experience in an epilepsy center or epilepsy clinic	13/91 (14.3%)	12/69 (17.4%)
Constant update with scientific publications and international and national guidelines	11/91 (12.0%)	13/69 (18.9%)
YES activities rated as the most important in educational growth^a^		
Facilitating participation to national and international congresses and courses by providing bursaries	34/104 (32.7%)	20/71 (28.1%)
Facilitating training experiences at third‐level centers in Italy and abroad	30/104 (28.8%)	22/71 (31.0%)
Planning educational meetings in which scholars are asked to present their own cases	18/104 (17.3%)	19/71 (26.8%)
Organizing mentorship programs	13/104 (12.5%)	2/71 (2.8%)
Facilitating learning by dissemination of scientific papers and books or manuals on epilepsy	9/104 (8.7%)	8/71 (11.3%)
YES activities rated as the most important for educational growth^a^		
Facilitating participation to national and international congresses and courses by providing bursaries	34/104 (32.7%)	20/71 (28.1%)
Facilitating training experiences at third‐level centers in Italy and abroad	30/104 (28.8%)	22/71 (31.0%)
Planning educational meetings in which scholars are asked to present their own cases	18/104 (17.3%)	19/71 (26.8%)
Organizing mentorship programs	13/104 (12.5%)	2/71 (2.8%)
Facilitating learning by dissemination of scientific papers and books or manuals on epilepsy	9/104 (8.7%)	8/71 (11.3%)
Preferred topic(s) for a future YES congress^a^		
Diagnostic pathways in epilepsy	25/75 (33.4%)	20/69 (29.0%)
Pharmacological management	22/75 (29.3%)	19/69 (27.5%)
Precision medicine (i.e., genetics, advanced imaging, immunological markers)	22/75 (29.3%)	18/69 (26.1%)
Epilepsy surgery	4/75 (5.3%)	7/69 (10.1%)
Leadership	2/75 (2.7%)	1/69 (1.5%)
Diagnosis communication in epilepsy	NA	3/69 (4.3%)
Other	NA	1/69 (1.5%)
Expectations toward YES‐I mission^a^		
Supporting education and career development	33/79 (41.8%)	52/71 (73.2%)
Financing congresses or bursaries/fellowships	21/79 (26.5%)	8/71 (11.3%)
Increasing visibility of the young LICE members	19/79 (24.1%)	6/71 (8.5%)
Increasing registration of young epileptologists to LICE	4/79 (5.1%)	4/71 (5.6%)
“I have no idea”	2/79 (2.5%)	1/71 (1.4%)

^a^ More than one selected option.

#### 3.1.1 Demographics and professional experience

In both surveys, female respondents slightly prevailed: 38 of 60 (63.3%) in 2019 and 42/69 (60.9%) in 2022 (Table 1 and Figure 1).

Responders were mainly from northern (25/60, 41.7% in 2019 and 47/69, 68.1% in 2022) or central Italy (23/60, 38.3% in 2019 and 14/69, 20.3% in 2022). Southern Italy (7/60, 11.7% in 2019 and 7/69, 9.7% in 2022) and major islands (5/60, 8.3% in 2019 and 1/69, 1.9% in 2022) were less represented.

One out of 60 (1.7%) responders in 2019 and 2/69 (2.9%) in 2022 were 20–25 years of age, 23/60 (38.3%) in 2019 and 22/69 (31.9%) in 2022 were 25–30, 27/60 (45.0%) in 2019 and 24/69 (34.8%) in 2022 30–35, while 9/60 (15.0%) in 2019 and 21/69 (30.4%) in 2022 were 35–40. In 2019, 37/60 (61.6%) were trainees, 16/60 (26.7%) worked as consultants, 6/60 (10%) were PhD students or research fellows, and 1/60 (1.7%) was a biologist. In 2022, 43.5% (30/69) worked as consultants, 40.6% (28/69) as trainees, 13.0% (9/69) as PhD students or research fellows, and 2.9% (2/69) were medical students.

Length of experience in epilepsy management was: <1 year in 21.4% (15/69) in 2019 versus 17% (10/60) in 2022, 2–5 years in 30.4% (21/69) in 2019 versus 46.6% (28/60) in 2022, and 5–10 years in 17.4% (12/69) in 2019 versus 26.6% (16/60) in 2022. It was more than 10 years in 14.65% (10/69) in 2019 versus 5% (3/60) in 2022.

While in 2019 the majority of responders (56.7%) worked as adult epileptologists, in 2022, the majority (50.7%) worked in pediatric epileptology. Epilepsy represented between 50% (23/60) and 75% (21/60) of total clinical practice for the majority (44/60, 73.3% in 2019; 46/69, 66.7% in 2022).

#### 3.1.2 Representativeness of the samples

By comparing demographic data from the total of YES‐I members and responders to the surveys, we did not find any significant differences in gender, age groups, or years of experience in the field of epilepsy, confirming that our samples are representative of the YES‐I population.

## 4 SURVEYS

### 4.1 Educational needs and areas of improvement

In 2019, more than one preference was given to this question (number of replies: 81). Participants indicated that they would need to improve in clinical neurophysiology (27/81 replies, 33.3%) followed by genetics (21/81, 25.9%) and neuroradiology (20/81, 24.7%). Drug therapy and clinical management received 8 (9.9%) and 5 (6.2%) preferences, respectively. In 2022, the most selected field was pharmacology (20/69, 29.0%), followed by neuroradiology (18/69, 26.1%), clinical neurophysiology (15/69, 21.7%), and genetics (14/69, 20.3%). Clinical management was the least selected (2/69, 2.9%) (Table 1 and Figure 2).

In 2022, we further evaluated the highest educational needs within each field (Figure 3).

Regarding pharmacology, 31/69 (44.9%) selected pharmacokinetics and drug‐to‐drug interactions, 16/69 (23.2%) correct second‐line drug after first‐line drug failure, 8/69 (11.6%) management of status epilepticus, 6/69 (8.7%) therapeutic approach to the first seizure, 4/69 (5.8%) management of adverse events, and 4/69 (5.8%) “other.”

Regarding neuroradiology, 33/69 (47.8%) indicated recognition of cortical malformations, 15/69 (21.7%) identification of other causes of structural epilepsy, 13/69 (18.8%) selecting the correct MRI sequences according to the clinical hypothesis, and 6/69 (8.7%) indications for high‐field MRI.

Within clinical neurophysiology, 20/69 (29%) selected specific patterns of status epilepticus, 18/69 (26.1%) recognition of physiological variants versus abnormal patterns, 16/69 (23.1%) recognition of specific neurophysiological patterns in the main epileptic syndromes, 11/69 (15.9%) abnormal sleep patterns, and 4/69 (5.9%) “other.”

Within genetics, 23/69 (33.3%) expressed their preference for selection of genetic tests according to patient's history, 22/69 (31.9%) genotype–phenotype correlations, 13/69 (18.8%) laboratory techniques, 9/69 (13%) precision medicine, and 2/69 (3%) “other.”

Regarding clinical aspects, 28/69 (40.6%) indicated the classification of genetic epilepsies, 19/69 (27.5%) differential diagnosis, 11/69 (15.9%) status epilepticus classification, and 5/69 (7.3%) management of the first seizure, while 6/69 (8.7%) selected “other.”

### 4.2 Experience

In 2019, more than one preference was given to this question (number of replies: 63).

The field in which responders thought they had the widest experience was diagnosis and differential diagnosis for 63.5% (40/63), EEG for 19.0% (12/63), and management of pharmacological therapies for 17.5% (11/63).

In the 2022 survey, responders only selected one response. Of 69 responders, 34 (49.3%) chose diagnosis and differential diagnosis, 20 (29.0%) EEG, 8 (11.6%) therapeutic management, and 7 (10.1%) “other” (Table 1 and Figure 2).

### 4.3 Courses and congresses

In 2019, more than one preference was given to this question (number of replies: 73).

Participants indicated that attendance to courses and congresses was not always possible due to their costs (31/73 replies, 42.5%), and might be difficult due to work organizational issues (22/73, 30.1%). For 9/73 (12.3%), participation to congresses and courses was part of the training school program, while for 7/73 (9.6%) it was not and thus not encouraged. For 4/73 (5.5%) of participants, attendance to courses and congresses was possible by relying on research funding or grants.

In 2022, there was a rise in the rate of participants who indicated that courses and congresses were difficult to attend due to organizational issues (35/69, 50.7%). The number of responders focusing their attention on costs declined (16/69, 23.2%). The remaining replies were similar to 2019, with 11/69 (15.9%) where courses and congresses attendance was part of their educational program versus 4/69 (5.8%) where it was not and thus not encouraged. Finally, for 3/69 (4.3%), attendance to courses and congresses was possible by relying on research funding or grants (Table 1 and Figure 2).

### 4.4 Preferred ways to improve knowledge and professional skills

In 2019, more than one preference was given to this question (number of replies: 91).

Responders thought that an educational and working experience at a third‐level center would be most useful, either abroad (28.6%, 26/91 replies in 2019 and 24.6%, 17/69, in 2022) or in Italy (26.4%, 24/91 in 2019 and 27.5%, 19/69, in 2022). A working experience in an epilepsy center or epilepsy clinic was less frequently chosen (14.3%, 13/91 in 2019 and 17.4%, 12/69 in 2022). In 2019, the least selected option was the constant update with scientific publications and international and national guidelines (12.0%, 11/91 responses), which received 13/69 preferences (18.9%) in 2022. Of note, in 2022 the least favored option (8/69, 11.6%) was attending congresses and courses, which on the contrary was the third most selected option in 2019 (18.7%, 17/91) (Table 1 and Figure 2).

### 4.5 Preferred topic for a future YES‐I congress

More than one preference was given to this question in 2019 (number of replies: 75). Preferences included diagnostic pathways in epilepsy (25/75 responses, 33.4% in 2019; 20/69, 29.0% in 2022), followed by pharmacological management (22/75, 29.3% in 2019; 19/69, 27.5% in 2022) and precision medicine (22/75, 29.3% in 2019; 18/69, 26.1% in 2022). In 2019, 5.3% (4/75) gave also preference to epilepsy surgery (vs. 10.1%, 7/69 in 202) and 2.7% (2/75) to leadership (vs. 1.5%, 1/69 in 2022). In 2022, diagnostic communication in epilepsy was also proposed and received 3/69 preferences (4.3%) (Table 1 and Figure 2).

In the 2022 survey, further questions on each topic were added (Figure 4).

Regarding diagnostic pathways in epilepsy, 20/69 (29.0%) of responders chose EEG as a diagnostic topic for a future YES‐I congress, followed by diagnostic evaluation of genetic epilepsies (12/69, 17.4%), differential diagnoses (12/69, 17.4%), evaluation of status epilepticus (12/69, 17.4%), management of the first seizure (9/69, 13.0%), and neuroradiology (4/69, 5.8%).

On drug therapy, preferred topic included pharmacokinetics and drug‐to‐drug interactions (24/69, 34.8%) and therapeutic switch to a second antiseizure medication after first‐drug failure (23/69, 33.3%). Management of adverse events, status epilepticus or the first seizure were chosen by 7/69 each (10.1%).

Regarding precision medicine, the most relevant topic was treatable forms of epilepsy for 24/69 (34.8%), followed by immunological markers (18/69, 26.1%), genetic epilepsies (15/69, 21.7%), and advanced neuroimaging (9/69, 13.0%), while 3/69 (4.4%) chose “other”.

Regarding epilepsy surgery, 30/69 (43.5%) chose indications to epilepsy surgery and evaluation of epilepsy surgery candidates as their preferred topic, 19/69 (27.5%) neuroimaging techniques, 9/69 (13%) EEG monitoring, 8/69 (11.6%) stereo‐EEG, and 3/69 (4.4%) neuropsychological evaluation.

### 4.6 YES‐I activities and mission

In 2019, 104 preferences were gathered. In 2022, these were 71.

In 2019, the YES activities rated as the most important for educational progress included facilitating participation to national and international congresses and courses by providing bursaries (32.7%, 34/104 preferences) and facilitation of training experiences at third‐level centers in Italy and abroad (28.8%, 30/104). Planning educational meetings in which scholars present their own cases was considered important in 17.3% of cases (18/104), while mentorship programs in 12.5% (13/104). Ongoing learning by dissemination of scientific papers, books, and manuals on epilepsy was chosen by 8.7% of participants (9/104).

In 2022, the most important activity was facilitation of fellowships at third‐level centers, abroad, and/or in Italy (22/71, 31.0%). It was followed by facilitating attendance to courses and congresses (20/71, 28.1%), educational events in which attendees would present and discuss cases (19/71, 26.8%), promoting education by broadcasting the latest scientific publications and academic books (8/71, 11.3%), and implementation of mentoring programs (2/71, 2.8%) (Figure 3).

Regarding expectations toward YES‐I mission, 79 preferences were expressed in 2019 and 71 in 2022. No change was observed between the two surveys. Supporting education and career development was the most favored option (41.8%, 33/79 responses in 2019 and 73.2%, 52/71 in 2022). Financial support toward congress attendance with bursaries (26.5%, 21/79 in 2019 and 11.3%, 8/71 in 2022) and increasing visibility of the young LICE members (24.1%, 19/79 in 2019 and 8.5%, 6/71 in 2022) followed. Four out of 79 (5.1%) of responders in 2019 and 4/71 (5.6%) chose increased participation to LICE activities, while 2/79 (2.5%) in 2019 and 1/71 (1.4%) had “no idea” (Figure 5).

In the 2019 survey, among final suggestions, responders highlighted the need for an online platform enabling constant exchange of clinical and EEG information, journal clubs, discussion of clinical cases, and national and international research collaborations.

## 5 CORRELATIONS BETWEEN SELECTED VARIABLES AND EXPRESSED NEEDS

Data from the 2022 survey were further analyzed in order to establish if selected variables (gender, field of clinical practice, and professional role) were associated with different educational or career development needs. Responses did not differ according to gender.

For the remaining two variables, we are reporting only results reaching statistical significance.

Epileptologists working with pediatric patients chose more often neuroradiology (13/35, 37.1%), drug therapy (12/35, 34.3%), and neurophysiology (8/35, 22.8%), as the fields where they feel the highest need for improvement. Adult epileptologists expressed most preferences for improvement in genetics (7/23, 30.4%) and drug therapy (7/23, 30.4%) (*p* = 0.018). Among clinical topics, adult neurologists considered diagnostic approach to genetic epilepsies as the main area needing improvement (13/23, 56.5%), while epileptologists working with children chose differential diagnosis (13/35, 37.1% vs. 4/23, 17.4%) and diagnostic approach to the first seizure (4/35, 11.4%; vs. 0/23, 0.0%) (*p* = 0.027).

Referring to genetics, epileptologists working with children chose genotype–phenotype correlations (13/35, 37.1% vs. 5/23, 21.7%) as the area of highest need for improvement. Those working with adults gave more preference to genetic investigations to request based on patient's history (12/23, 52.2% vs. 10/35, 28.6%) (*p* = 0.034). The biggest difference in preferences was seen in precision medicine, indicated by 7/33 (21.21%) of child epileptologists versus 1/23 (4.35%) adult epileptologists. Conversely, regarding experience, the main differences were found in the management of drug therapies, chosen mostly by adult epileptologists (7/23, 30.4%, vs. 1/35, 2.8%), while preference of childhood epileptologists was more shifted toward EEG (13/35, 37.1%, vs. 3/23, 13.0%) (*p* = 0.007).

In the clinical field, while the majority of trainees needed to improve more on differential diagnosis (12/28, 42.8%), both consultants (17/30, 56.7%) and trainees (9/28, 32.1%) indicated diagnostic approach to genetic epilepsies (*p* = 0.004). In genetics, both consultants (13/30, 43.3%) and trainees (10/28, 35.7%) chose selection of genetic tests based on patient's history, while genotype–phenotype correlation was the preferred choice for PhD students and research fellows (4/9, 44.4%) (*p* = 0.022).

Participation to courses and congresses was not always possible for trainees due to their costs (14/28, 50.0%), while the main issue for consultants (22/30, 73.3%) was organization issues at work (*p* = 0.005).

Regarding YES‐I activities, consultants mainly indicated that YES‐I should organize educational meetings in which attendants present their own cases (12/30, 40.0%). It should also promote professional update by spreading relevant scientific publications and books (5/30, 16.7%; vs. 1/28, 3.6%, trainees and 1/9, 11.1%, PhD students and research fellows). For trainees, the most important YES‐I activity is facilitating participation of young epileptologists to courses and congresses through bursaries (14/28, 50.0%). PhD students and research fellows chose implementation of educational experiences in third‐level centers in Italy and abroad (3/9, 33.3%). They were also the only responders to choose mentoring programs (2/9, 22.2%) (*p* = 0.003).

## 6 DISCUSSION

With the two surveys, the YES‐I Education and Career Task Force investigated the needs and preferences of young Italian epileptologists regarding their professional education and career development. We obtained similar response rates in the two surveys (approximately 15%). We had the opportunity to evaluate our audience's views regarding their strongest areas of experience, areas of improvement, learning and education, interest in attending congresses and courses, and obstacles preventing participation, expectations toward YES‐I activities and mission.

No gender or geographical differences were found in participation between the two surveys. However, some differences can be highlighted. The cohort changed in that in 2019 the majority of responders worked in adult epileptology, while in 2022 the majority worked in pediatric epileptology. While in 2019 the main field needing improvement was clinical neurophysiology, in 2022 it was pharmacology. Barriers to attendance of courses and congresses changed because in 2022 there was an increase in the number of epileptologists indicating organizational issues and a decline in those selecting costs‐related issues. Strikingly, attendance of congresses and courses went from being the third most selected way to improve knowledge and professional skills in 2019 to become the least favored option in 2022. According to our survey results, strategies to promote attendance to congresses should include not only reducing the costs for junior delegates or offering bursaries and prizes (which is already part of LICE policies), but also making congresses and courses more engaging and interactive, or promoting hybrid (on‐site and online participation) in order to counteract organizational issues, which increased after the COVID‐19 pandemic. As previously suggested, conferences should target specific categories of health‐care providers and have a specific goal, mission, and vision encouraging the achievement of a well‐defined objective at the end of the meeting.^1^

Mentorship programs also declined from 12.5% in 2019 to 2.8% in 2022. In both surveys, participants indicated as the area of major experience diagnosis and differential diagnosis. Diagnostic pathways in epilepsy was the favored topic for a future YES‐I congress in both. Finally, despite working experience at a third level center was considered as the best way to improve professional skills in both surveys, while in 2019 experiences abroad prevailed over experiences in Italy, the opposite was true in 2022.

As, to our knowledge, this represents the first survey to address the perceived educational and career needs of young epileptologists, we are unable to directly compare our results with the literature. A recent paper focused on the educational needs of trainees with differing years of experience and backgrounds, chosen among medical specialties likely to get involved in the management of persons with epilepsy, including but not limited to neurology. This study was designed to investigate in which field's responders thought they most needed training. Intuitively, different specialties and different levels of expertise led to evolving educational needs, from basic to more advanced.^2^

In the absence of previous data in epileptology, we can propose partial comparison of our results with those of a survey by the American Headache Society, investigating the major issues among early career consultants and trainees. Data from this survey contrast our findings in that career planning, but also logistics and opportunities for involvement where chosen as the most pressing issues, while the identification of research as a top issue followed at the fourth position.^3^ However, not only differences in health systems organization between Italy and the United States, but also differences between subspecialties might account for these differences, possibly being headache medicine more clinic‐oriented and private practice‐oriented than epileptology.

By comparing the two surveys, administered 3 years apart and with the COVID‐19 pandemic in between, some of the results did not significantly change, first of all response rate and geographical distribution of responders. Female participants prevailed in both surveys, reflecting increasing representation of female doctors in younger age groups, a global trend confirmed by the Organisation for Economic Co‐operation and Development (OECD) (https://www.oecd.org/gender/data/the‐proportion‐of‐female‐doctors‐has‐increased‐in‐all‐oecd‐countries‐over‐the‐past‐two‐decades.html).

However, other results did change, probably because there are different cohorts that might be only partially overlapping and do not represent the whole cohort of young Italian epileptologists. We could not specifically evaluate the possible impact of the COVID‐19 pandemic in explaining some of the differences between the two survey results. They might be at least partially linked not only to the profound reorganizational efforts brought on by the pandemic on public health systems,^4^ but also to the effect of job role.

In Italy, the young members of the Società Italiana di Neurologia (SIN, Italian Neurology Society) (SIgN) investigated the impact of COVID‐19 pandemic on neurology training program in Italy, confirming the use of virtual platforms to deliver training schools lessons in 92% of cases, and relevant changes in clinical and research activities during residency programs,^5^ likely playing a role in our results.

A further survey from the same group investigated the future of neurology following COVID‐19 pandemic. Residents thought that the pandemic would lead to a reorganization of neurological activity, with an increase in outpatient assistance, a reduction in the number of available beds and a reduction in the quality of care.^6^

Interestingly, our survey participants did not seem to value “traditional” learning methods, and this seems to be especially true for trainees. This is confirmed by the low rating of scientific papers and epilepsy books or manuals dissemination by YES‐I as a valid contribution for educational growth. The increasing preference for alternative learning methods, including e‐learning^7^ or podcasting^8^ is also being pursued by scientific societies. For example, the European Association of Neurologists (EAN) developed an e‐learning platform during the COVID‐19 pandemic (https://www.ean.org/learn/elearning, last accessed June 23, 2023). Interactive educational and teaching methods can effectively improve learning.^9^, ^10^ An alternative explanation is that, although these methods are valued “per se,” expectations on YES‐I mission do not include promoting traditional learning strategies and materials, a need that might be adequately addressed elsewhere (i.e., specialty training schools, university, autonomous search, and word of mouth).

Expectations on the YES‐I role have not significantly changed between the two surveys. Young epileptologists expect YES‐I to support their education and career developments, to support bursaries and congress participation and to increase their visibility within LICE. Our audience is not currently interested in learning about leadership programs, while they seem to be keener to be assisted in shaping their career in terms of high quality clinical and research experiences.

Our study was mainly designed to implement bottom‐to‐top decision‐making and plan future YES‐I activities accordingly. Participation of trainees and young specialists in designing neurology training and practice has been previously advocated.^8^ By assessing the needs of young epileptologists, we were able to elaborate different projects, including webinars, podcasts, and mentor–mentee programs.

We assessed whether gender, field of clinical practice and job position have a role in our audience's preferences. We did not find a role of gender, suggesting that gender may affect negotiation, compensation, representation, career opportunities, and life–work balance,^11^ but not educational or career needs/preferences. Second, adult and child neurologists have different professional skills, reflected by their different preferences, to be taken into account when proposing scientific meetings, especially those with stronger educational aims. Finally, job position may determine differences at various levels. Expectations toward YES‐I mission and aims differ in that trainees would need financial support in joining courses and congresses, while young consultants would like to be offered educational meetings in which to actively discuss with senior staff. Finally, PhD students and research fellows are the ones giving more value to educational and job experiences at third level centers and mentoring programs. Although our survey was not designed to examine in more depth the reasons behind these preferences, it is possible to speculate that PhD students and young researchers might be interested in integrating basic science, clinical and translational research, and in obtaining excellent professional expertise. These data highlight the need to generate targeted educational offers and career‐shaping projects for trainees, early‐career consultants, and PhD students or research fellows.

The low response rate in both surveys represents a limitation of this study. However, percentages were calculated on the total number of YES‐I members and not on the number of delegates taking part to the congresses. Furthermore, by comparing the main demographic data of YES‐I members to those of responders to the two surveys, we did not find any statistically significant difference, confirming that our samples, although small, are representative of the whole groups of YES‐I members in the two studied periods (2019 and 2022). We could not perform further comparisons on additional data (i.e., job position or field of clinical practice) as these types of information were not available for the whole cohort of YES‐I members.

## 7 CONCLUSIONS

By these surveys, we collected useful suggestions on how to organize future YES‐I activities, namely by directly involving younger epileptologists in interactive case presentations and discussions, promoting higher levels of interaction between senior and junior LICE members, facilitating fellowships, and promoting bursaries. The preferred topic for a future YES‐I congress would be diagnostic pathways in epilepsy.

In conclusion, these surveys represent a first effort by YES‐I to investigate the needs of young Italian epileptologists. We aim to monitor these results by further surveys over time.

## AUTHOR CONTRIBUTIONS

All authors took part into experimental design, data acquisition, and interpretation of data; Jacopo Lanzone performed the statistical analysis; Carlotta Spagnoli wrote the first draft of the manuscript; Maddalena Duca, Veronica Pelliccia, Jacopo Lanzone, Silvia Masnada, Daniela Chiarello, Tommaso Lo Barco, Fedele Dono, and Bruna Nucera critically revised the article with respect to intellectual content; all authors approved the manuscript in its final version.

## FUNDING INFORMATION

No funding was secured for the completion of this manuscript.

## CONFLICT OF INTEREST STATEMENT

The authors declare that they have no conflict of interest to disclose. We confirm that we have read the Journal's position on issues involved in ethical publication and affirm that this report is consistent with those guidelines.

## Data Availability

Data are not publicly available, but they will be made available from the corresponding author upon reasonable request.
